# Water Cure

**Published:** 1876-02

**Authors:** 


### WATER CURE.

ONE of the most powerful of remedial means is the use of cold water—powerful for good or ill. Much of the prejudice existing against it is unjust, having arisen from its injudicious application by incompetent men. Any valuable remedy is liable to abuse. Beyond all question, *calomel*, in the estimation of the old school, is worth all the other remedies of Allopathic materia medica; but nine tenths of those who employ it, do so injudiciously, and one of the great reasons of this injudicious use, is in the fact that inconsiderate practitioners, living in one section of the country, have taken "*reported cases*" from other and distant sections for their guide.

So with the errors of water cure. Its wise and safe application consults the varying habits, temperaments, constitutions, and modes of life of those who employ it. The truly intelligent men who practise the water cure, owe it to the reputation of a useful remedy, to impress upon their younger brethren the value of a thoughtful discrimination in every case. A lady of unusual intelligence writes:

"I was so unfortunate as to be over-treated at a water cure. I believe the Doctor did his best to cure me, but the treatment was too powerful for a person the most marked feature of whose case has always been great depression of vital power. It produced entire sleeplessness. It was more. I was preternaturally awake. For four days and nights I did not lose my consciousness for a single moment. When, at the end of this time, and life was almost extinct, I would fall asleep, and for a week sleep some, after a fashion, then another of those terrible attacks of sleeplessness would come on, and run its course, no matter what was done. In this way I suffered for more than a year, and then I began to sleep better; but I am sure my system received a great shock, and I doubt if I ever sleep as well as other people. I have been obliged to give up cold bathing altogether. A single bath will deprive me of the power of sleeping. I now use tepid sponging every other day, with soap, and think it agrees with me."

We knew an estimable gentleman some years ago, of small vitality, and very feeble constitution. He could not keep warm. The cold water mania seized him at this time; he carried it to the greatest extremes, when chronic diarrhœa set in, and he died.

